# Whole-genome sequencing to establish relapse or re-infection with *Mycobacterium tuberculosis*: a retrospective observational study

**DOI:** 10.1016/S2213-2600(13)70231-5

**Published:** 2013-12

**Authors:** Josephine M Bryant, Simon R Harris, Julian Parkhill, Rodney Dawson, Andreas H Diacon, Paul van Helden, Alex Pym, Aziah A Mahayiddin, Charoen Chuchottaworn, Ian M Sanne, Cheryl Louw, Martin J Boeree, Michael Hoelscher, Timothy D McHugh, Anna L C Bateson, Robert D Hunt, Solomon Mwaigwisya, Laura Wright, Stephen H Gillespie, Stephen D Bentley

**Affiliations:** a Wellcome Trust Sanger Institute, Hinxton, UK; b Division of Pulmonology, University of Cape Town, Cape Town, South Africa; c DST/NRF Centre of Excellence for Biomedical Tuberculosis Research, MRC Centre for Molecular and Cellular Biology, Division of Molecular Biology and Human Genetics, Stellenbosch University, Tygerberg, South Africa; d South African Medical Research Council and KwaZulu Research Institute for TB and HIV, Durban, South Africa; e Institute of Respiratory Medicine, Kuala Lumpur, Malaysia; f Chest Disease Institute, Muang, Nothaburi, Thailand; g Clinical HIV Research Unit, Helen Joseph Hospital, Westdene, Johannesburg, South Africa; h Madibeng Centre for Research, Brits, South Africa; i Radboud MD University Nijmegen Medical Centre/UCCZ Dekkerswald, Nijmegen, Netherlands; j Department of Infectious Diseases and Tropical Medicine, Klinikum, Ludwig-Maximilians-University, Munich, Germany; k DZIF German Centre for Infection Research, Munich, Germany; l Centre for Clinical Microbiology, Royal Free Campus, University College London, London, UK; m School of Medicine, University of St Andrews, St Andrews, UK

## Abstract

**Background:**

Recurrence of tuberculosis after treatment makes management difficult and is a key factor for determining treatment efficacy. Two processes can cause recurrence: relapse of the primary infection or re-infection with an exogenous strain. Although re-infection can and does occur, its importance to tuberculosis epidemiology and its biological basis is still debated. We used whole-genome sequencing—which is more accurate than conventional typing used to date—to assess the frequency of recurrence and to gain insight into the biological basis of re-infection.

**Methods:**

We assessed patients from the REMoxTB trial—a randomised controlled trial of tuberculosis treatment that enrolled previously untreated participants with Mycobacterium tuberculosis infection from Malaysia, South Africa, and Thailand. We did whole-genome sequencing and mycobacterial interspersed repetitive unit-variable number of tandem repeat (MIRU-VNTR) typing of pairs of isolates taken by sputum sampling: one from before treatment and another from either the end of failed treatment at 17 weeks or later or from a recurrent infection. We compared the number and location of SNPs between isolates collected at baseline and recurrence.

**Findings:**

We assessed 47 pairs of isolates. Whole-genome sequencing identified 33 cases with little genetic distance (0–6 SNPs) between strains, deemed relapses, and three cases for which the genetic distance ranged from 1306 to 1419 SNPs, deemed re-infections. Six cases of relapse and six cases of mixed infection were classified differently by whole-genome sequencing and MIRU-VNTR. We detected five single positive isolates (positive culture followed by at least two negative cultures) without clinical evidence of disease.

**Interpretation:**

Whole-genome sequencing enables the differentiation of relapse and re-infection cases with greater resolution than do genotyping methods used at present, such as MIRU-VNTR, and provides insights into the biology of recurrence. The additional clarity provided by whole-genome sequencing might have a role in defining endpoints for clinical trials.

**Funding:**

Wellcome Trust, European Union, Medical Research Council, Global Alliance for TB Drug Development, European and Developing Country Clinical Trials Partnership.

## Introduction

Tuberculosis is a chronic pulmonary infection that can recur after initial successful treatment. The prevalence of recurrent tuberculosis worldwide is thought to be low; WHO has estimated that, of the 6·2 million cases of tuberculosis in 2011, roughly 5% were recurrences.^1^ Recurrence can happen by two means: relapse of the primary infection that treatment has failed to eradicate or re-infection with an unrelated exogenous strain. Molecular genotyping techniques—such as IS6110 fingerprinting, mycobacterial interspersed repetitive unit-variable number of tandem repeat (MIRU-VNTR), and spoligotyping—are based on the detection of differences in the number or location of particular genetic sequences or signatures and have enabled researchers to assess the proportion of recurrences caused by re-infection.

Both mixed infections (the presence of more than one strain)^2^ and exogenous re-infection occur often and the rate of re-infection is related to tuberculosis infection density.^3^ In high incidence regions—eg, Cape Town, South Africa—re-infection is common, with one study classifying 77% of recurrences as re-infection.^4^ Higher rates of re-infection are associated with HIV infection.^5^ For example, in India, an estimated 88% of recurrences in HIV-positive patients were caused by re-infection compared with only 9% in HIV-negative patients.^6^

The resolution of established molecular typing techniques is limited by the highly monomorphic nature of the *Mycobacterium tuberculosis* population. Whole-genome sequencing of *M tuberculosis* enables isolates to be differentiated with much greater resolution, providing insights into evolution,^7^ epidemiology,^8^, ^9^ and mutation rate.^9^, ^10^, ^11^ To date, whole-genome evolutionary analyses have been limited because of the diversity of proline–glutamate (PE) and proline–proline–glutamate (PPE) gene families. Genes in these families have been excluded from analyses because of their high guanine and cytosine content and their repetitive nature, which make sequencing and genome assembly difficult. Despite accounting for almost 10% of the coding capacity of the genome,^12^ the function of PE and PPE genes is unknown. There is a high degree of diversity between isolates of *M tuberculosis*, for both individual PE and PPE genes^13^, ^14^ and the gene families as a whole.^15^ Exclusion of these genes in previous analyses might have resulted in underestimates of genetic diversity. PE and PPE are probably involved in interactions with the human immune system, and perhaps immune evasion. However, no direct evidence exists of within-patient antigenic variation of these genes.

We investigated the use of whole-genome sequencing to distinguish the source of relapse in patients infected with *M tuberculosis*.

## Methods

### Study design and participants

We analysed the first 50 paired isolates available from participants in the multicentre REMoxTB drug trial (registered with ClinicalTrials.gov, number NCT00864383). These pairs consisted of an initial isolate and second isolate from after week 17 of treatment, taken by sputum sample from patients with relapse or bacteriological failure. As described previously,^16^ eligible patients were adults diagnosed with previously untreated, drug-sensitive, smear-positive, pulmonary tuberculosis without severe comorbidities. HIV-positive patients with a CD4 count below 250 cells per μL or those already taking antiretroviral treatment were excluded. All participants were treated for tuberculosis for 26 weeks with one of three regimens lasting 4 months or 6 months and containing rifampicin, isoniazid, ethambutol, pyrazinamide, moxifloxacin, or placebo. The total observation period was 18 months, including treatment and follow-up. All patients, researchers, and staff involved were masked to treatment allocation. The study was approved by local and national ethics committees and regulatory authorities.^16^ Participants provided informed consent. Consent was written unless the participant was illiterate, in which case witnessed oral consent was used. Strains and clinical data presented in this report were used according to the approvals and consent procedures of REMoxTB.

### Procedures

We assessed clinically relevant cases—ie, patients with treatment failure or recurrent disease. We differentiated between treatment failure and recurrence by reviewing complete case history, including all culture results and all clinical information available. Cases were defined as single isolated positive culture when a positive culture was followed by at least two negative cultures without re-treatment having been initiated by a physician and the patient remaining free of symptoms throughout the remainder of follow-up.

We took a 10 μL loop from a Löwenstein-Jensen medium slope and, after heat killing, extracted DNA first by digestion with lysozyme and proteinase K, solubilised by detergents sodium dodecyl sulphate and cetrimonium bromide, followed by chloroform isopropanol extraction.^17^ We did MIRU-VNTR by amplification of 24 loci, as described previously.^18^

We constructed sequencing libraries in two multiplex pools of 62 and 38 isolates with each isolate uniquely tagged. The DNA polymerase KAPA HiFi (KapaBiosystems, Woburn, MA, USA) provides good coverage for regions of the genome with high guanine and cytosine content,^19^ so we used it to generate all the sequencing libraries in our study. The pools were subjected to paired-end sequencing on a single lane of the HiSeq platform (Illumina, San Diego, CA, USA) with a read length of 100 base pairs. Reads are deposited in the European Nucleotide Archive (accession number ERP001037).

For analysis of single nucleotide polymorphisms (SNPs), we mapped reads to a corrected version of the H37Rv reference genome^20^ with SMALT (version 0.5.8) and identified SNPs as previously described.^11^, ^21^ We built a maximum likelihood phylogenetic tree based on the variable positions with RAxML (version 7.0.4).^22^ SNPs between recurrence pairs were recorded only if the base in both isolates passed quality checks.^11^, ^21^ Any SNPs in the PE and PPE genes that differed between the relapse pairs were discounted. Differences between the relapse pairs were checked manually against the raw sequencing data. Mixed-base positions were identified as sites where more than one base had been identified in a single sample, where each allele was supported by at least 5% of reads (minimum read depth of four). We included only positions without strand bias (p>0·05),^23^ that had coverage within the normal range, that had mapping quality score greater than 50, and that had base quality scores greater than 30. These cutoffs accord with those that would be applied to high quality SNPs and were prespecified to minimise false positives.^21^ Sites within 200 base pairs of other heterozygous sites were discounted because of the possibility that they might have been caused by a mapping error.

Mapping PE and PPE genes is prone to errors and can result in artifacts because sequences are not unique; therefore, an assembly approach is needed. We analysed the genes with de-novo assembly using Velvet (1.2.03).^24^ SNPs, insertions, and deletions were identified from pairs of isolates from relapse patients and manually assessed. We mapped raw sequence reads back onto the assembly to identify possible errors (appendix).

### Role of the funding source

The sponsor of the study had no role in study design, data collection, data analysis, data interpretation, or writing of the report. The corresponding author had final responsibility for the decision to submit for publication. JMB, SRH, SDB, and JP had access to all the raw genomics data, SHG had access to all the clinical data, SHG, TDMcH, ALCB, RDH, LW, and SM had access to the microbiological data.

## Results

**Figure 1:**
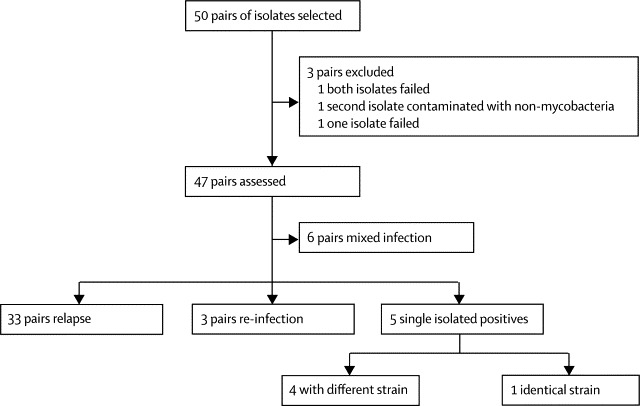
Distribution of the case outcomes for study patients based on sequencing quality data, sequence comparison, and clinical evaluation

We did whole-genome sequencing for paired samples from 50 patients (11 of whom were HIV seropositive). None of the isolates showed resistance to study drugs, as measured by mycobacterial growth indicator tube susceptibility testing or genome sequence. For 96 of the samples (47 patient-pairs plus two pairs where a result was not obtained from one sample and another pair where both isolates failed to sequence; figure 1) we obtained an average coverage of 120-fold. We excluded four samples because of poor coverage or contamination with a non-mycobacterial source (figure 1). Based on the 10 354 variable positions, we built a maximum likelihood phylogeny, which showed four globally recognised lineages (appendix).

**Figure 2:**
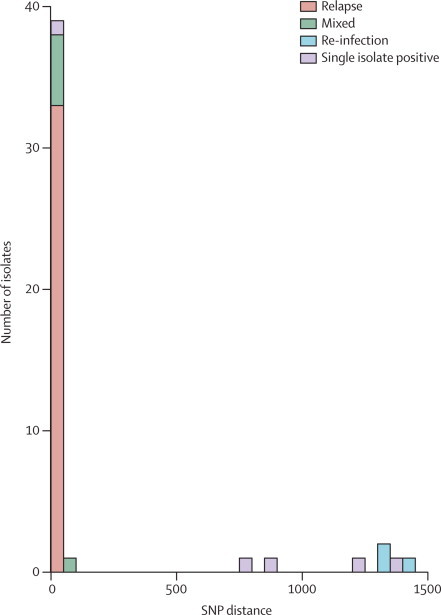
Pair-wise distances between pairs of isolates from the same patient For each patient pair, the calculated pair-wise distance is based on the number of high quality base differences between the samples.

We classed a pair as a mixed infection if one of the isolates had more than 80 heterogeneous base pairs. Discounting mixed infections, differences of SNPs between the relapse and re-infection pairs was large (figure 2), with a clear distinction between pairs with little difference (≤6 SNPs), and those with a high difference (≥1306). Walker and colleagues report^9^ that within-patient diversity does not generally exceed 14 SNPs, with most patients having fewer than five differences between initial and later isolates.^9^ Thus, we are confident that pairs with few differences are relapses, and those with many differences are re-infection.

We identified relapses in 33 of 47 patients (70%; table 1), with pairs differing by a mean of 0·47 SNPs (SD 1·21) and most (n=27) having no polymorphisms. Three pairs (7%)—which all had more than 1306 differences—were re-infections. All three re-infections involved isolates belonging to different lineages: either the Euro-American or east Asian type. The mean SNP distance between the re-infection isolates was 1355, which is greater than the mean pairwise distance between all isolates in the whole dataset (972).

**Table 1:** Whole-genome sequencing results and patient information, by patient ID

	**Time between episodes (weeks)**	**SNP distance**	**Site**
**Relapses**			
4	48	6	Stellenbosch, SA
5	26	0	Stellenbosch, SA
6	26	0	Cape Town, SA
7	36	0	Stellenbosch, SA
9	26	0	Stellenbosch, SA
11	26	0	Stellenbosch, SA
12	36	0	Stellenbosch, SA
13	36	1	Stellenbosch, SA
16	26	0	Cape Town, SA
17	26	0	Cape Town, SA
18	48	0	Stellenbosch, SA
19	36	0	Stellenbosch, SA
20	48	0	Stellenbosch, SA
21	36	1	Stellenbosch, SA
22	26	0	Cape Town, SA
24	36	0	Cape Town, SA
25	48	0	Cape Town, SA
26	36	0	Cape Town, SA
27	36	0	Stellenbosch, SA
28	36	0	Stellenbosch, SA
29	36	0	Cape Town, SA
30	26	0	Stellenbosch, SA
31	26	0	Stellenbosch, SA
32	26	0	Durban, SA
33	60	0	Durban, SA
34	36	0	Durban, SA
40	36	0	Durban, SA
41	28	2	Johannesburg, SA
43	36	1	Brits, SA
44	48	0	Brits, SA
47	17	0	Kuala Lumpur, MY
48	36	0	Nonthaburi, TH
49	36	2	Nonthaburi, TH
**Re-infections**			
10	48	1419	Cape Town, SA
14	60	1340	Cape Town, SA
35	17	1306	Durban, SA
**Mixed infections**			
2	36	64	Cape Town, SA
8	26	48	Stellenbosch, SA
23	36	0	Stellenbosch, SA
42	37	1	Brits, SA
45	17	1	Kuala Lumpur, MY
50	36	0	Nonthaburi, TH
**Single isolated positives**			
3	36	3	Stellenbosch, SA
15	26	1364	Stellenbosch, SA
36	28	898	Durban, SA
37	48	1207	Durban, SA
38	60	767	Durban, SA

SNP=single nucleotide polymorphism. MY=Malaysia. SA=South Africa. TH=Thailand.

For 87 samples, we identified fewer than 40 heterogeneous sites across the genome, which was probably caused by mapping error. However, for seven pairs, more than 80 sites were identified (appendix) and were manually inspected for mixed bases at lineage-defining positions, or where SNPs had been identified in the other isolate of the pair.^25^ Six patients had evidence of a mixed infection, of whom four were mixed in the first sample of the patient's pair. The isolates were heterogeneous at positions where an SNP was identified in the second sample, indicating that the initial sample contained a strain found in the second isolate plus a sequence from another lineage. Two patients had evidence of two distinct strains only in the second isolate (appendix), one of which was the same strain as in the initial sample, which could be interpreted as relapse and superinfection. An additional sample also had evidence of a mixed population, but was defined clinically as a single isolated positive.

Five cases were defined clinically as single isolated positives. These cases had been defined by whole-genome sequencing as a different strain (n=3), a mixed infection (n=1), and relapse (n=1). The relapse strain differed by three single nucleotide polymorphisms, suggesting that this case was a true relapse and not cross-contamination.

**Figure 3:**
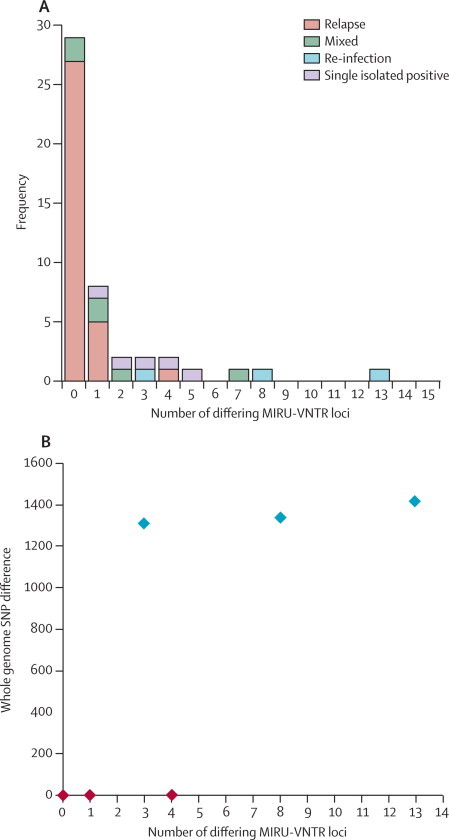
MIRU-VNTR loci differing between pairs of isolates from the same patient (A) Number of loci differing between pairs of isolates from the same patient with relapse, re-infection, mixed infection, and single isolated positives identified by whole genome sequencing. (B) Comparison of differences detected by whole genome sequence data and MIRU-VNTR; red=relapse, blue=re-infection. MIRU-VNTR=mycobacterial interspersed repetitive unit-variable number of tandem repeat.

Table 2 shows the different results obtained with 24 locus MIRU-VNTR typing and whole-genome sequencing. The three pairs identified as re-infection by whole-genome sequencing differed by 3–13 loci. As expected most relapse cases (n=27) had an identical MIRU-VNTR type, but six differed by one or more loci (figure 3). Six cases that were identified as possible mixed infections by whole-genome sequencing were identified as re-infections (n=4) or relapse (n=2) by MIRU-VNTR.

**Table 2:** Designation of recurrence strains by MIRU-VNTR, by whole-genome sequencing designation

	**MIRU-VNTR designation**	
	Relapse	Re-infection
Relapse (n=33)	27	6
Mixed infection (n=6)	2	4
Re-infection (n=3)	0	3
Single isolated positive (n=5*)	0	5

MIRU-VNTR=mycobacterial interspersed repetitive unit-variable number of tandem repeat.

* Includes one isolate identified as a relapse (three single nucleotide polymorphisms) and one sample that is mixed according to whole-genome sequencing.

Of 18 SNPs identified between the relapse pairs, 13 were non-synonymous. These mutations were in genes that encode proteins with various functions (appendix); for two cases, the genes were involved in survival during oxidative stress. We identified two insertion–deletion differences that resulted in frame-shifts within protein-coding regions.

Use of Kappa HiFi improved coverage of PE and PPE gene families by an average of 27% in all subfamilies compared with the standard protocol (appendix). Overall, 86% of genes (82% PPE, 97% non-PGRS PE, 84% PE–PGRS) were assembled, which enabled 82% of genes to be compared between the 24 pairs (appendix). For the 24 pairs for which the PE and PPE genes were assembled, we detected no additional variants in these regions (appendix).

## Discussion

Our study is the first to our knowledge to assess whole-genome sequencing in the context of relapse and re-infection with tuberculosis. Clinical trials for tuberculosis are based on the assumption of clonal infection. Thus, when disease re-occurs, it is assumed to be a relapse and this is the primary endpoint for many studies underway at present. Spoligotyping,^26^ insertion sequence typing (IS6110),^27^ and MIRU-VNTR^18^ have all been used to show that re-occurring strains are highly dissimilar, suggesting exogenous re-infection.^3^, ^4^, ^6^ However, these techniques lack the resolution needed for more detailed investigation of re-infection,^28^ and the different techniques sometimes provide incongruous data.^29^ The ability to accurately distinguish relapse from re-infection is of critical importance to understanding tuberculosis epidemiology and for choosing endpoints for clinical trials and patient management. Our findings provide proof of principle that when a patient is infected with a single strain, whole-genome sequencing can unequivocally distinguish relapse and re-infection (panel).

Panel **Research in context**

**Systematic review**

We searched PubMed with the terms “whole genome sequencing”, “mixed infection”, and “tuberculosis typing” for studies published up to June 1, 2013. We included reports published in English on the basis of the relevance of the method used and tuberculosis epidemiological situation described. 14 reports were included. These reports show that re-infection was common in the study population based on the measure of MIRU–VNTR and that, according to whole-genome sequencing data, patients have few single nucleotide polymorphisms (usually fewer than six) when they are involved in person-to-person transmission events.^9^, ^11^ No previous groups have used whole genome sequencing in the context of a tuberculosis clinical trial.

**Interpretation**

Whole genome sequencing provides a new approach to assess strain evolution and the availability of pairs from a well-characterised sample provided an unrivalled opportunity to evaluate the role of this technology in a clinical trial setting. Our study adds to the understanding of events by showing that whole genome sequencing can unequivocally identify relapse (<six single nucleotide polymorphisms). We also show that some true relapses would have been misclassified as recurrence with a different strain (re-infection) had MIRU-VNTR been the only technique used. At least 11% of infections were with two genetically distinct strains. These findings have important implications for the design of clinical trials, especially in relation to how endpoints are defined, particularly for mixed infection and laboratory cross-contamination. The large genetic distance between re-infection strains requires the new strain to be significantly different genetically to the original strain. Future studies are needed to test this finding because it would suggest that re-infection could be caused by the absence of immune protection, which could be important for vaccine design.

The presence of different numbers of copies of loci in recurrence pairs that had no detectable differences in SNPs, suggests that previous estimates of re-infection rates might have been too high, because many studies use a cutoff of one MIRU-VNTR locus.^6^, ^30^ Variation in the MIRU-VNTR loci between isolates differing by less than five SNPs has also been reported previously.^9^ Likewise, for one pair separated by more than 760 SNPs, we detected a difference at only one MIRU-VNTR locus. Overall, the genetic diversity of relapse and re-infection cases overlapped when assessed with MIRU-VNTR loci, but were clearly separated when we used whole-genome screening. Our data suggest that the relationship between the two methods for the rate of variation generated is non-linear and that the regular accumulation of SNPs is a more reliable marker of genetic relatedness between strains.

The different classification of outcome by MIRU-VNTR will have a substantial effect on interpretation and design (particularly sample size) of clinical trials. 18% of cases were misclassified as relapse—the primary endpoint in many clinical trials—indicating the need to use whole-genome sequencing to classify these outcomes more accurately. Use of whole-genome sequencing will determine the endpoint more accurately, which should result in smaller sample sizes and reduced costs of studies.

During clinical trials of tuberculosis treatment, a single strain is usually isolated during patient selection, which is assumed to be representative. Although the bacteriological methods of our study were focused on the isolation of a single strain, six patients had mixed infection. Four of these patients would have been classified as re-infected using MIRU-VNTR typing alone. This high number of mixed infections in endemic areas accords with previous findings.^2^ Additionally, we could not always amplify all loci, especially in cases of mixed infection. These data suggest that further studies are needed using whole-genome sequencing to understand how common mixed infection is in different settings and its effect on the evolution of drug resistance. The implication for clinical trials is that more strenuous efforts should be made to identify mixed infection that could be incorrectly identified as re-infection. This might require sequencing of multiple colonies or non-colony-purified cultures of multiple samples.

In a previous study,^31^, ^32^ 405 single isolated positive samples were identified from 37 429 samples taken from 2133 patients in four clinical trials with no clinical evidence of relapse. Without effective typing methods available, positive results were ascribed to clerical error, cross-contamination, or as arising from the patient's lesions.^31^ Experiments reported at the same time showed that transfer from positive to negative samples occurred in 0 of 825 (0%) samples in a laboratory in London and in 28 of 2165 (1·3%) samples in Kampala.^32^ Of the five cases we identified as single isolated positives, four were probably caused by cross-contamination and one provides evidence for the first time that positive cultures originating from the patient's own infection can be cultured and the patient's infection resolved without further chemotherapy. Cross-contamination is a well-recognised challenge in mycobacteriology laboratories. It occurs in less than 1% of positive samples overall with more than half of laboratories achieving a proportion of less than 2·5%.^32^, ^33^ In clinical trials, investigators should ensure that adequate molecular methods are used to correctly identify the origin of single isolated positive samples.

Our findings reaffirm the high genomic stability previously reported^9^, ^11^ for *M tuberculosis* in the context of transmission, in which the rate of genetic turnover of *M tuberculosis* was 0·3–0·5 SNPs per genome per year. Previous studies^15^ have reported more variation in the PE and PPE genes than we detected, but many genes—particularly those belonging to the PE–PGRS subfamily—were not included in previous whole-genome sequencing studies. We were able to assemble 86% of the PE and PPE genes in a set of relapse pairs including 84% of the PE–PGRS genes. This shows that diversity in these genes—discounted from most previous studies of the *M tuberculosis* genome—can be captured with short reads. We detected no variation—ie, SNPs, insertions, or deletions—between any of the relapse pairs. The lack of diversity in these genes suggests that variation within patients is low; indicating that such diversity might not be the mechanism of immune escape, as previously postulated.^15^ However, these genes might generate diversity over longer timescales or differentially regulated expression of these genes might enable immune escape.

We have shown that a large phylogenetic distance separates re-infection pairs belonging to different lineages and distinct from relapse cases. As far as we know, this finding has not been previously reported, probably because of the limitations of earlier genotyping technologies, which include high levels of incongruence.^29^ This finding should be followed up in subsequent studies because it implies that re-infection requires the new strain to be significantly genetically different to the originally infecting strain. If confirmed, it could indicate that re-infection occurs because primary infection does not provide sufficient immune protection against distantly related strains, which is of importance for future vaccine design.
