# Supplementary material for: Whole-genome sequencing to establish relapse or re-infection with Mycobacterium tuberculosis: a retrospective observational study
Source: Lancet Respir Med. 2013 Dec;1(10):786–92. doi: 10.1016/S2213-2600(13)70231-5 (PMC3861685; doi:10.1016/S2213-2600(13)70231-5)
Supplement: Supplementary appendix [file mmc1.pdf]

THE LANCET Respiratory Medicine

Supplementary appendix

This appendix formed part of the original submission and has been peer reviewed. We post it as supplied by the authors.

Supplement to: Bryant JM, Harris SR, Parkhill J, et al. Whole-genome sequencing to establish relapse or re-infection with *Mycobacterium tuberculosis*: a retrospective observational study. *Lancet Respir Med* 2013; published online Nov 21. http://dx.doi.org/10.1016/S2213-2600(13)70231-5.

Supplementary materials

Supplementary methods

PE and PPE gene family analysis

Analysis of the PE and PPE genes was carried out for 48 of the isolates corresponding to 24 relapse pairs. Due to the limitations of mapping, the genes were analysed via *de novo* assembly. Velvet was used to assemble the reads with scaffolding enabled. ABACAS[1] was then used to order these contigs with respect to the reference genome.[2] Gaps were filled with Ns and unmapped contigs appended to the end of the assembly. Raw reads were mapped back to the assembly to correct possible assembly errors. Sequences were extracted from the assemblies using an in-house script that uses a simulated PCR approach where upstream and downstream 'primer' sequences are specified, which were in non-repetitive regions outside the PE/PPE genes being analysed. Alignments were made using Muscle[3] for each gene for the 48 isolates. SNP and InDel differences were identified between the relapse pairs using an in-house script, and manually assessed.

Supplementary Figure 1 . Distribution of removed sample pairs. The diagram outlines the reasons that three sample pairs were excluded from the relapse/re-infection evaluation. The two isolates that passed the quality filters were, however, included in the phylogenetic analysis.

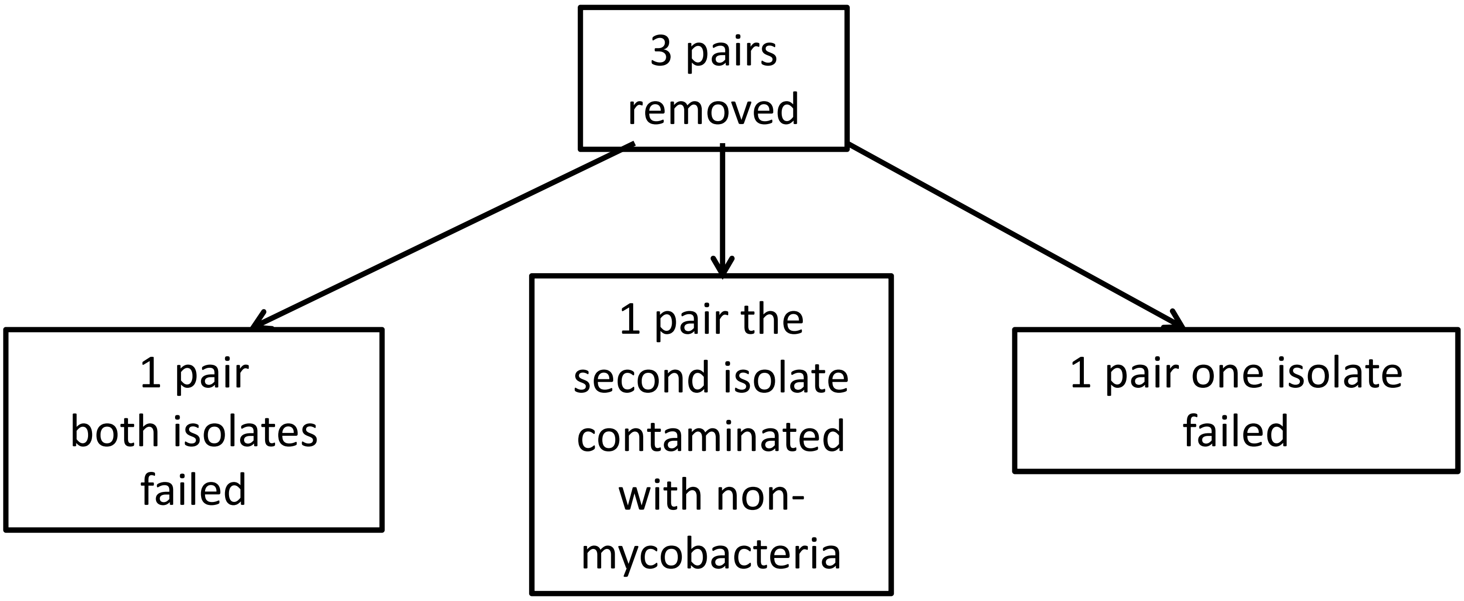

Supplementary Figure 2. Maximum likelihood tree. The tree was built on the 10,354 SNPs detected with 100 bootstrap replicates using RAxML [4]. Re-infection, relapse and mixed infection cases are indicated in red, blue and grey respectively. Isolates in black are those either suspected to be single isolated positives, unpaired isolates due to sequencing failures or the reference genome used for mapping.

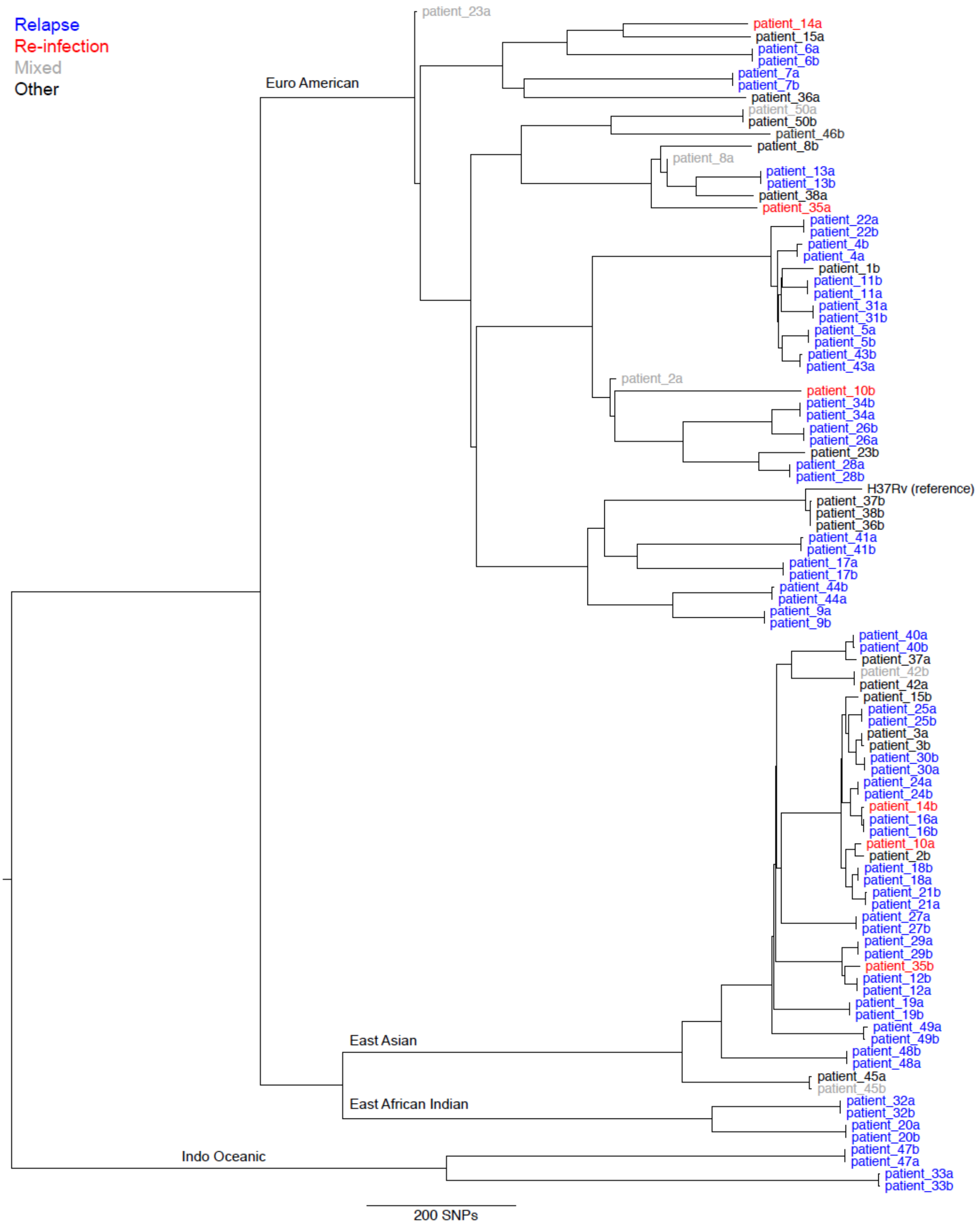

Supplementary Figure 3. Number of high quality heterogeneous SNPs identified in the samples. Those marked in grey are samples classed as mixed and subjected to further investigation.

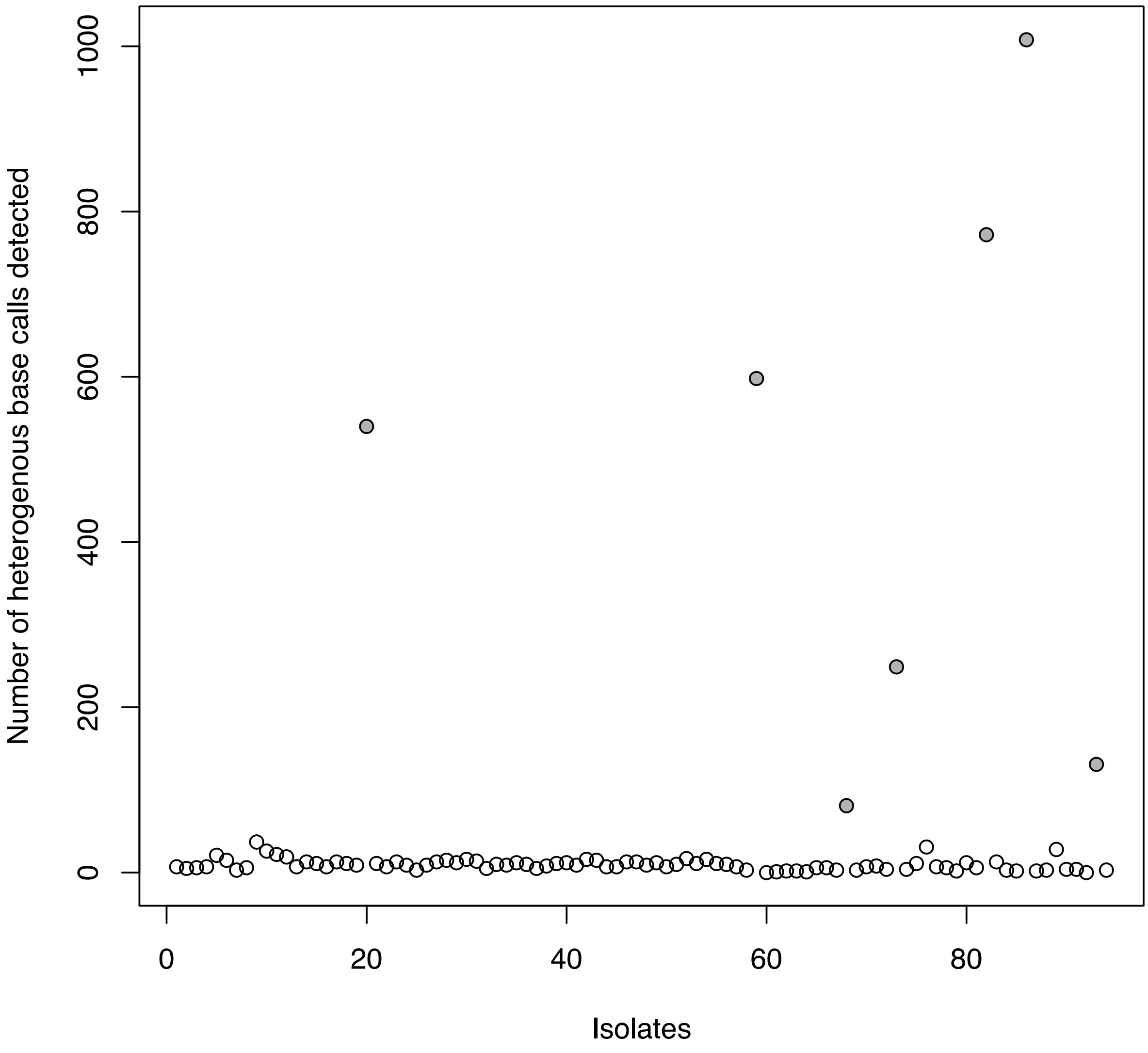

Supplementary Figure 4. Coverage of PE and PPE genes. A: Comparison of reads per kilobase per million reads (RPKM) for different gene classes and different library preparation enzymes. Comparison isolate obtained from from Casali *et al*.[5] Example showing relationship between coverage and GC content, and its improvement with Kapa HiFi. Figure adapted from Artemis.[6]

A

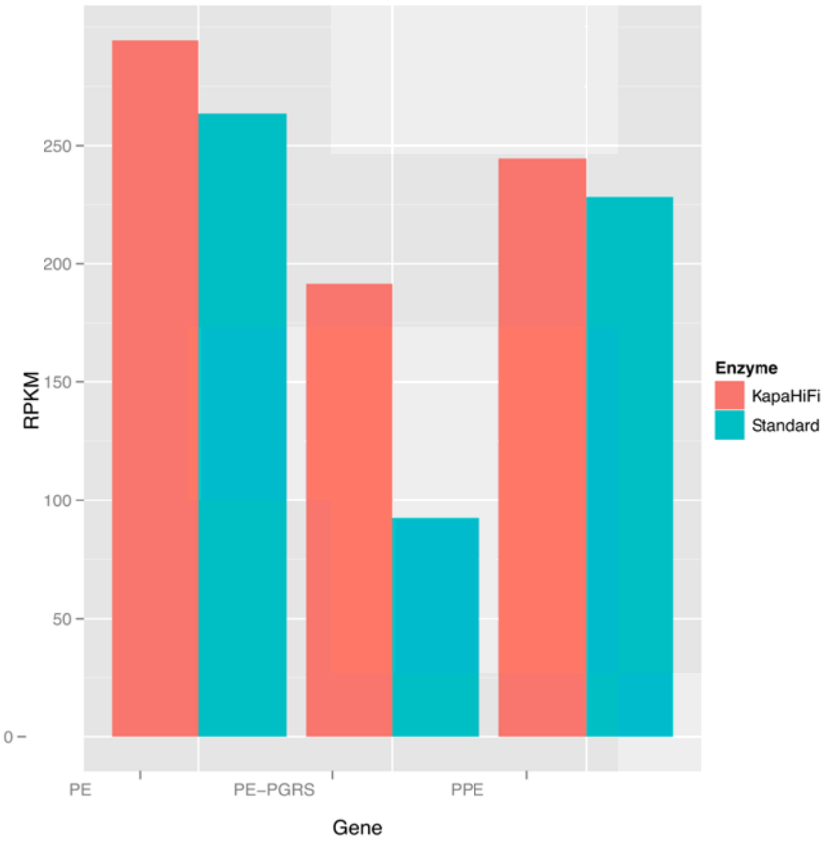

B

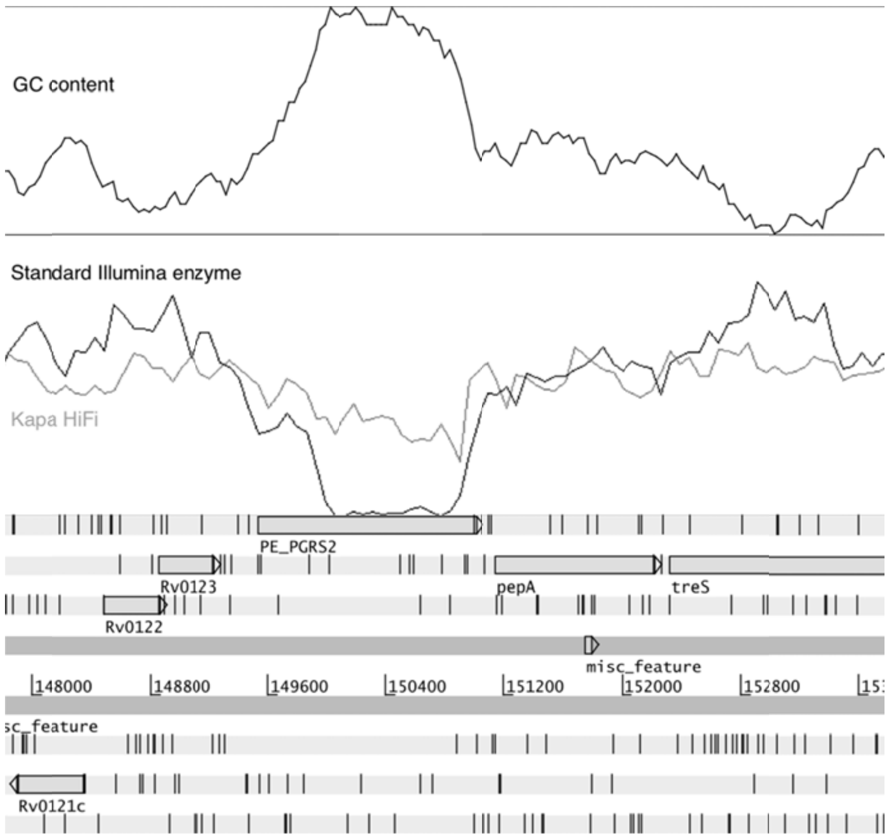

GC content
Standard Illumina enzyme
Kapa HiFi
PE_PGRS2
Rv0123
pepA
treS
Rv0122
misc_feature
148000
148800
149600
150400
151200
152000
152800
sc_feature
Rv0121c

Supplementary Table 1: SNPs identified between relapse pairs

Patient	Type	Systematic ID, gene name	Function	Virulence relevance
3	NS SNP	Rv1129c	Possible transcriptional regulator protein	Involved in stress pathways [7]
3	NS SNP	Rv2397c, cysA1	Sulfate-transport ATP-binding protein ABC transporter	
3	NS SNP	Rv1830	Conserved hypothetical protein	
4	NS SNP	Rv0083	Probable oxidoreductase	
4	NS SNP	Rv0105c, rpmb1	Probable rpmB1, 50S ribosomal protein	
4	NS SNP	Rv0726c	Conserved hypothetical protein	
4	NS SNP	Rv0800, pepC	Aminopeptidase I	
4	NS SNP	Rv3734c	Conserved hypothetical protein	
13	NS SNP	Rv3303c, lpdA	Dihydrolipoamide dehydrogenase	Protects against oxidative stress [8]
21	NS SNP	Rv1086	Chain Z-isoprenyl diphosphate synthase	
41	NS SNP	Rv1384, carB	Carbamoyl-phosphate synthase large chain	
41	NS SNP	Rv3294c	Conserved hypothetical protein	
49	NS SNP	Rv1533	Conserved hypothetical protein	
42	Frameshift deletion	Rv3307, deoD	Purine nucleoside phosphorylase	
44	Frameshift insertion	Rv3851	Possible membrane protein	

Supplementary Table 2: Number of PE and PPE genes assembled. Although most genes were successfully assembled, for a minority the results were poor. Most could be attributed to common deletions such as PPE57, PPE58 and PPE59 which are flanked by IS sequences, and PPE38 which is a hotspot for IS6110 integration.[9]

Gene	Number of isolates for which is was assembled (total=48)	Gene	Number of isolates for which is was assembled (total=48)	Gene	Number of isolates for which is was assembled (total=48)
PE1	48	PE_PGRS1	48	PPE1	48
PE2	48	PE_PGRS2	48	PPE2	48
PE3	48	PE_PGRS3	7	PPE3	48
PE4	48	PE_PGRS4	40	PPE4	48
PE5	48	PE_PGRS5	48	PPE5	48
PE6	48	PE_PGRS6	47	PPE7	33
PE7	48	PE_PGRS7	48	PPE9	48
PE8	48	PE_PGRS8	48	PPE10	48
PE9	48	PE_PGRS9	44	PPE11	48
PE10	48	PE_PGRS10	44	PPE12	48
PE11	48	PE_PGRS11	48	PPE13	45
PE12	48	PE_PGRS13	48	PPE14	48
PE13	48	PE_PGRS14	48	PPE15	48
PE14	48	PE_PGRS15	48	PPE16	26
PE15	48	PE_PGRS16	48	PPE17	48
PE16	48	PE_PGRS17	7	PPE18	13
PE17	48	PE_PGRS18	19	PPE19	16
PE18	46	PE_PGRS19	36	PPE20	48
PE19	48	PE_PGRS20	17	PPE21	48
PE20	48	PE_PGRS21	46	PPE22	48
PE22	48	PE_PGRS22	38	PPE23	48
PE23	48	PE_PGRS23	48	PPE24	33
PE24	48	PE_PGRS24	48	PPE25	9
PE25	48	PE_PGRS25	48	PPE26	46
PE26	48	PE_PGRS26	48	PPE27	0
PE27A	39	PE_PGRS27	10	PPE28	48
PE27	48	PE_PGRS28	10	PPE29	48
PE29	9	PE_PGRS29	48	PPE30	48
PE31	48	PE_PGRS30	48	PPE31	48
PE32	48	PE_PGRS31	48	PPE32	48
PE33	48	PE_PGRS32	48	PPE33	48
PE34	48	PE_PGRS33	48	PPE34	20
PE35	48	PE_PGRS34	48	PPE35	48
PE36	48	PE_PGRS35	48	PPE36	48
		PE_PGRS36	48	PPE37	48
		PE_PGRS37	48	PPE38	6
		PE_PGRS38	48	PPE39	48
		PE_PGRS39	48	PPE40	14
		PE_PGRS40	48	PPE41	48
		PE_PGRS41	48	PPE42	48
		PE_PGRS42	48	PPE43	48
		PE_PGRS43	48	PPE44	48

PE_PGRS44	48	PPE45	48
PE_PGRS45	13	PPE46	9
PE_PGRS46	48	PPE47	20
PE_PGRS47	48	PPE49	44
PE_PGRS48	48	PPE50	26
PE_PGRS50	33	PPE51	48
PE_PGRS51	48	PPE52	48
PE_PGRS52	45	PPE53	20
PE_PGRS53	45	PPE54	5
PE_PGRS54	18	PPE55	43
PE_PGRS55	11	PPE56	43
PE_PGRS58	47	PPE57	21
PE_PGRS57	0	PPE58	34
PE_PGRS59	48	PPE59	20
PE_PGRS60	48	PPE60	39
PE_PGRS61	48	PPE61	48
PE_PGRS62	48	PPE62	48
PE_PGRS63	48	PPE63	48
wag22	19	PPE64	48
		PPE65	48
		PPE66	46
		PPE68	48
		PPE69	46

	Indo-oceanic (3 920 109; G>T)	East Asian (1 834 177; A>C)	East African Indian (301 341; C>A)	Euro-American (3 326 554; C>A)	West African 1 (1 377 185; C>G)	West African 2 (2 427 828; C>G)
2a	0	0·16	0	0·84	0	0
2b	0	1	0	0	0	0
8a	0	0	0	1*	0	0
8b	0	0	0	1	0	0
23a	0	0·39	0	0·7	0	0
23b	0	0	0	1	0	0
42a	0	1	0	0	0	0
42b	0	0·96†	0	0	0	0
45a	0	1	0	0	0	0
45b	0	0·93	0	0·08	0	0
50a	0·26	0	0	0·69	0	0
50b	0	0	0	1	0	0

We identified lineage-specific SNPs according to Stucki and colleagues.[10] Frequencies are the proportion of reads that match the base that defines the lineage. *Composed of two Euro-American strains divergent by at least 132 SNPs in a 0·5 mix. †Composed of a Typical Beijing isolate identical to 42a (95%) plus an Atypical Beijing strain (5%).[11] Manual inspection of 42b also shows reads matching the Atypical strain (approximately 2%). SNP=single nucleotide polymorphism.

Table 3: **Proportion of reads matching lineage-defining single nucleotide polymorphisms identified in patients for who mixed infection is suspected, by patient sample**

References supplementary material

1. Assefa S, Keane TM, Otto TD, Newbold C, Berriman M. ABACAS: algorithm-based automatic contiguation of assembled sequences. Bioinformatics. 2009; **25**(15): 1968-9.
2. Casali N, Nikolayevskyy V, Balabanova Y, Ignatyeva O, Kontsevaya I, Harris SR, et al. Microevolution of extensively drug-resistant tuberculosis in Russia. Genome Res. 2012; **22**(4): 735-45.
3. Edgar RC. MUSCLE: multiple sequence alignment with high accuracy and high throughput. Nucleic Acids Res. 2004; **32**(5): 1792-7.
4. Stamatakis A. RAxML-VI-HPC: maximum likelihood-based phylogenetic analyses with thousands of taxa and mixed models. Bioinformatics. 2006; **22**(21): 2688-90.
5. Casali N, Nikolayevskyy V, Balabanova Y, Ignatyeva O, Kontsevaya I, Harris SR, et al. Microevolution of extensively drug-resistant tuberculosis in Russia. Genome Res. 2012; **22**(4): 735-45.
6. Carver T, Harris SR, Berriman M, Parkhill J, McQuillan JA. Artemis: An integrated platform for visualisation and analysis of high-throughput sequence-based experimental data. Bioinformatics. 2011.
7. Pang X, Vu P, Byrd TF, Ghanny S, Soteropoulos P, Mukamolova GV, et al. Evidence for complex interactions of stress-associated regulons in an mprAB deletion mutant of Mycobacterium tuberculosis. Microbiology. 2007; **153**(Pt 4): 1229-42.

8. Akhtar P, Srivastava S, Srivastava A, Srivastava M, Srivastava BS, Srivastava R. Rv3303c of Mycobacterium tuberculosis protects tubercle bacilli against oxidative stress in vivo and contributes to virulence in mice. Microbes Infect. 2006; **8**(14-15): 2855-62.
9. McEvoy CR, Cloete R, Muller B, Schurch AC, van Helden PD, Gagneux S, et al. Comparative analysis of Mycobacterium tuberculosis pe and ppe genes reveals high sequence variation and an apparent absence of selective constraints. PLoS One. 2012; **7**(4): e30593.
10. Stucki D, Malla B, Hostettler S, Huna T, Feldmann J, Yeboah-Manu D, et al. Two new rapid SNP-typing methods for classifying Mycobacterium tuberculosis complex into the main phylogenetic lineages. PLoS One. 2012; **7**(7): e41253.
11. Schurch AC, Kremer K, Warren RM, Hung NV, Zhao Y, Wan K, et al. Mutations in the regulatory network underlie the recent clonal expansion of a dominant subclone of the Mycobacterium tuberculosis Beijing genotype. Infect Genet Evol. 2011; **11**(3): 587-97.
